# Computer-Assisted Pterygium Screening System: A Review

**DOI:** 10.3390/diagnostics12030639

**Published:** 2022-03-05

**Authors:** Siti Raihanah Abdani, Mohd Asyraf Zulkifley, Mohamad Ibrani Shahrimin, Nuraisyah Hani Zulkifley

**Affiliations:** 1 Faculty of Humanities, Management and Science, Universiti Putra Malaysia (Bintulu Campus), Bintulu 97008, Sarawak, Malaysia; raihanah.abdani@siswa.ukm.edu.my (S.R.A.); ibrani@upm.edu.my (M.I.S.); 2 Department of Electrical, Electronic and Systems Engineering, Faculty of Engineering and Built Environment, Universiti Kebangsaan Malaysia, Bangi 43600, Selangor, Malaysia; 3 Community Health Department, Faculty of Medicine and Health Sciences, Universiti Putra Malaysia, Serdang 43400, Selangor, Malaysia; hanizulkifley@moh.gov.my

**Keywords:** pterygium assessment, eye disease screening, deep learning, classification, semantic segmentation

## Abstract

Pterygium is an eye condition that causes the fibrovascular tissues to grow towards the corneal region. At the early stage, it is not a harmful condition, except for slight discomfort for the patients. However, it will start to affect the eyesight of the patient once the tissues encroach towards the corneal region, with a more serious impact if it has grown into the pupil region. Therefore, this condition needs to be identified as early as possible to halt its growth, with the use of simple eye drops and sunglasses. One of the associated risk factors for this condition is a low educational level, which explains the reason that the majority of the patients are not aware of this condition. Hence, it is important to develop an automated pterygium screening system based on simple imaging modalities such as a mobile phone camera so that it can be assessed by many people. During the early stage of automated pterygium screening system development, conventional machine learning techniques such as support vector machines and artificial neural networks are the de facto algorithms to detect the presence of pterygium tissues. However, with the arrival of the deep learning era, coupled with the availability of large training data, deep learning networks have replaced the conventional networks in screening for the pterygium condition. The deep learning networks have been successfully implemented for three major purposes, which are to classify an image regarding whether there is the presence of pterygium tissues or not, to localize the lesion tissues through object detection methodology, and to semantically segment the lesion tissues at the pixel level. This review paper summarizes the type, severity, risk factors, and existing state-of-the-art technology in automated pterygium screening systems. A few available datasets are also discussed in this paper for both classification and segmentation tasks. In conclusion, a computer-assisted pterygium screening system will benefit many people all over the world, especially in alerting them to the possibility of having this condition so that preventive actions can be advised at an early stage.

## 1. Introduction

**Figure 1:**
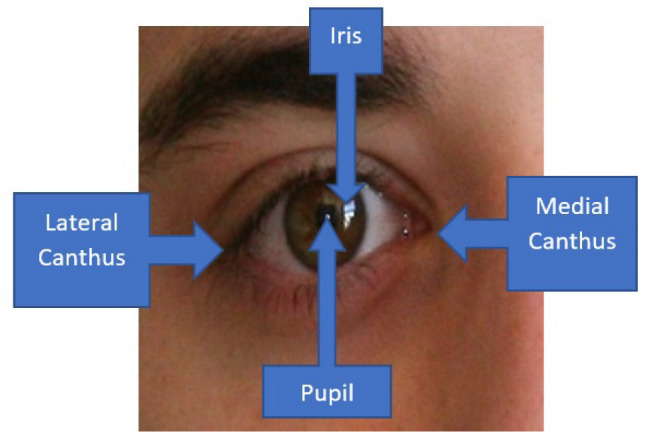
Medial canthus and lateral canthus of the eye.

Pterygium or also known as surfer’s eye, is a condition in which there is an overgrowth of fibrovascular tissues that originate primarily from the conjunctiva at the medial canthus region [1]. For more severe cases of pterygium, the condition develops from both the medial and lateral canthus regions, as shown in Figure 1. Initially, the abnormal tissues will involve the conjunctiva over the sclera, whereby the disease will encroach towards the corneal region once it becomes more severe. It is a noncancerous or benign type of tissue abnormality that usually has a hedge or kite shape [2]. Generally, it is often treated as a minor issue unless the abnormal tissues start to encroach upon the corneal region, which will consequently block light from coming into the macula region, whereby the eyesight will start to deteriorate. In the early stage, it can cause irritation and astigmatism, and hence cause discomfort to the patients [3]. Therefore, it is crucial to screen the condition at the early stage so that preventative actions can be advised properly to reduce and, eventually, to stop the growth of the abnormal tissues [4].

**Figure 2:**
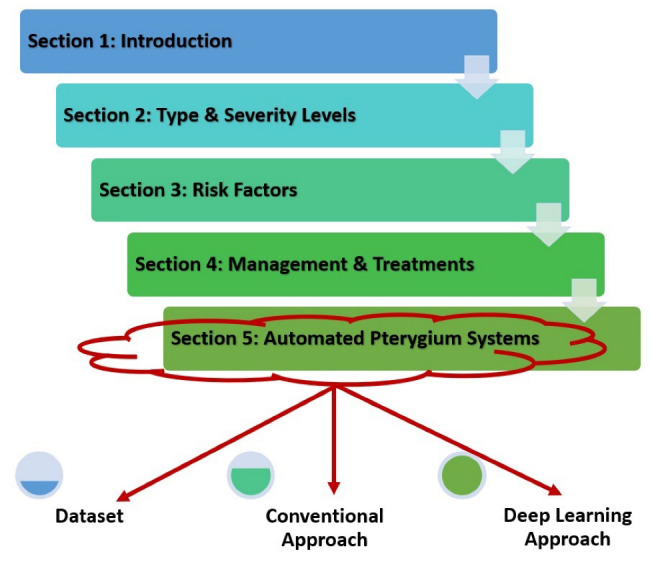
General information flow of this review paper.

This review paper is organized into six sections that start with a basic introduction to the pterygium condition. Then, the following Section 2 discusses the type and severity level of the pterygium condition, followed by risk factors for pterygium in Section 3. After considering the possible reasons for developing pterygium, Section 4 explains the management and treatment procedures of this condition, which are mainly divided into early and late stages. Section 5 summarizes the main state-of-the-art methods in automated pterygium screening systems for both classification and segmentation tasks, which are further divided into several subsections that include the dataset used to fit the model, conventional machine learning methodology, and deep learning techniques applied in the screening system. The final Section 6 concludes the paper, followed by a few suggestions on future research work to further improve the performance of a computer-assisted pterygium screening system. The general information flow of this paper is shown in Figure 2.

## 2. Pterygium Type and Severity Levels

**Figure 3:**
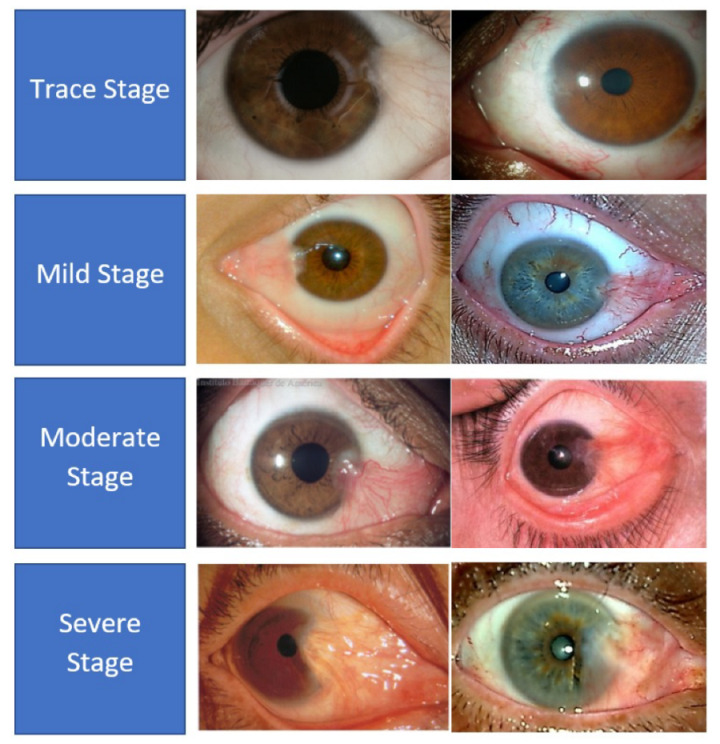
Samples of pterygium-infected tissues according to the severity level.

There are two types of pterygium, atrophic and progressive, which are differentiated mainly by the growth patterns of the abnormal tissues [5]. In the progressive type of pterygium, the fleshy tissues will continually grow towards the corneal region with more vascularity. On the other hand, the atrophic pterygium is a lighter version between these two types, whereby the tissues will stop growing after the initial growth stage. Medical practitioners have grouped the severity levels of pterygium into four classes, which are trace, mild, moderate, and severe cases [6]. In the trace stage, the pterygium tissues rarely reach the corneal region and they appear translucent, with very few dilated blood vessels. During the mild stage, the density of the dilated blood vessels increases significantly, which makes the pterygium tissues resemble a pinkish kite pattern. Then, in the moderate cases, the dilated blood vessels become denser, and hence, the kite pattern tissues appear more reddish rather than pinkish in color. In the last stage or the severe case, the red tissues can cover the majority of the white areas of the eyes, and dense networks of blood vessels can be observed clearly. Figure 3 shows some samples of the severity levels of pterygium.

## 3. Pterygium Risk Factors

According to the meta-analysis of 20 studies by Liu et al. [3], the global prevalence of pterygium is around 10%, with a slightly higher occurrence among males compared to females. A few risk factors are associated with pterygium and the most popular among them is frequent exposure to ultraviolet radiation [7]. A few papers have discussed the possible risk factors among pterygium patients in China, including the Inner Mongolian region, Shandong province, and Dali city. According to Zhong et al. [8], the risk factors of pterygium among the residents of Yunnan province are older age, lack of formal education, and presence of outdoor work. The work in [9] then pointed out two additional risk factors, which are the usage of hats and sunglasses. However, they did not find any significant correlation between the prevalence of pterygium between women and men. Another study that focused on the Inner Mongolian region of China found that the main risk factors of pterygium are frequent outdoor activities and older age [10]. Contrary to previous findings, they did not find any associated risk factor between educational level and pterygium. In addition to these studies from China, Malekifar et al. [11] analyzed the risk factors for pterygium in Iran. They found two unique risk factors, which are a family history of pterygium and severe blepharitis. Another study from the Asian region was performed in [12] and they found that a significant risk factor could be observed among males compared to females. This finding is in contrast to the studies in [8,9], whereby the authors concluded that systemic factors will not induce the pterygium condition. Besides the above, another study [13] from Gambella, Ethiopia agreed with the previously observed major risk factors, such as exposure to sunlight and outdoor activities. However, they also found that males have greater risk of being affected by pterygium compared to females, which supports the findings of Cahjucom-Uy et al. [12]. The findings from a study in the American region [14] also supported the general conclusion of previous works, relating the pterygium risk to the educational factor and exposure to sunlight. Table 1 summarizes the main risk factors of pterygium and the experimental details.

## 4. Pterygium Management and Treatment

**Figure 4:**
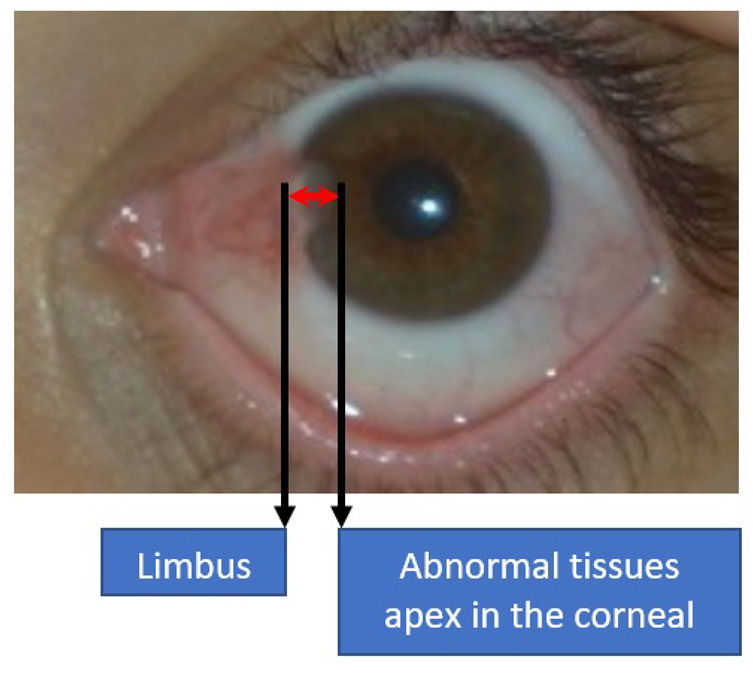
Measurement distance between the limbus and the apex of abnormal tissues in the corneal region.

Generally, ophthalmologists have agreed that pterygium at the early stage is a minor concern, in which a short-term solution is usually advised through topical corticosteroid eye drops [2]. Therefore, a detailed history of the suspected pterygium patient will be analyzed first to identify any anomaly or other early indicators. It is important to distinguish the pterygium case from the ocular surface squamous neoplasia case, which can be distinguished through the period of the abnormal tissue growth [15]. At the early stage, precautions include ultraviolet-filtered sunglasses, lubricant eye drops, and anti-inflammatory eye drops. Meanwhile, for severe cases, surgery is the most viable option for treatment [16]. Some ophthalmologists will alert the patients if the pterygium tissues grow to more than 3 mm, which is the distance measured from the limbus to the apex of the corneal region, as shown in Figure 4. According to Aminlari et al. [17], three surgical procedures can be performed to remove the benign tissues, which include bare sclera excision, the conjunctival autograft technique, and amniotic membrane grafting. In [18], Janson and Sikder suggested additional three surgical procedures to treat pterygium, namely primary closure surgery, the conjunctival flap procedure, and a limbal conjunctival autograft.

## 5. Automated Pterygium Detection and Localization

Since only the most severe cases of pterygium will require corrective surgery, this condition needs to be detected at the early stage, especially during the trace and mild phases. During these early phases, simple medication such as eye drops can prevent the condition from becoming worse, hence avoiding the need for surgery. As a result, screening for pterygium is very important and needs to be made available to lower-income workers. Furthermore, pterygium’s main risk factor is a low educational level, which is common among workers who work outdoors and are frequently exposed to sunlight for long periods. Therefore, a simple screening system should be developed to enable everyone to perform self-screening simply by using a standard mobile phone camera. It is worth noting that the reviewed methods aim to detect and localize the pterygium cases without considering any possibility of recurrent cases. This is because the reviewed images and their labels do not contain enough information to determine whether the detected cases are new or recurrent ones. More labeling information is needed by ophthalmologists to further detect cases of repeated conditions. Taking motivation from this argument, many studies have been carried out on pterygium detection using anterior segment photograph images [19], which can also be captured using a standard mobile phone camera. However, the user then still needs to verify the presence of pterygium and its severity level with licensed medical practitioners. Hence, many researchers have embarked on automated pterygium detection and localization research. The goal of the detection module is to determine the presence of pterygium tissues in an image without knowing the severity level of the disease. The user will be notified of their likelihood of having the pterygium condition or not. As an extension to the detection module, the localization module is designed to provide the severity level information, whereby the size of the benign tissues is directly correlated with the pterygium stage. Two popular approaches to identify the size of the tissues are object detection and semantic segmentation methods. The following subsections will discuss the existing dataset available to train the computer-assisted system, followed by a discussion of the state-of-the-art automated detection and localization of pterygium.

### 5.1. Dataset

Any computer-assisted system that utilizes a supervised learning approach will require a reasonable number of data and their corresponding labels so that the model is able to learn the disease characteristics. In general, the conventional machine learning approach requires a lesser number of training data compared to the deep learning approach [20]. In addition, the data augmentation method can also be implemented to increase the variety of the training data, either through simple image transformation or synthetically generated images [21]. Moreover, the most basic technique to overcome the limitations in the training dataset is by using transfer learning, either by freezing the convolutional layers or retraining the whole network again. There are two main components that will be the main concern for an automated pterygium screening system, which are to detect the presence of pterygium in an image and to segment the pterygium tissues so that the disease severity level can be determined. Currently, there are two popular datasets that have been collected for classification purposes, which are Zaki et al.’s [19] and Fang et al.’s [22] datasets. Moreover, a dedicated dataset has been developed by Abdani et al. [7] for segmentation purposes that contains a combination of all severity levels. Cai et al. [23] go a step further by providing images for both classification and segmentation purposes.

**Figure 5:**
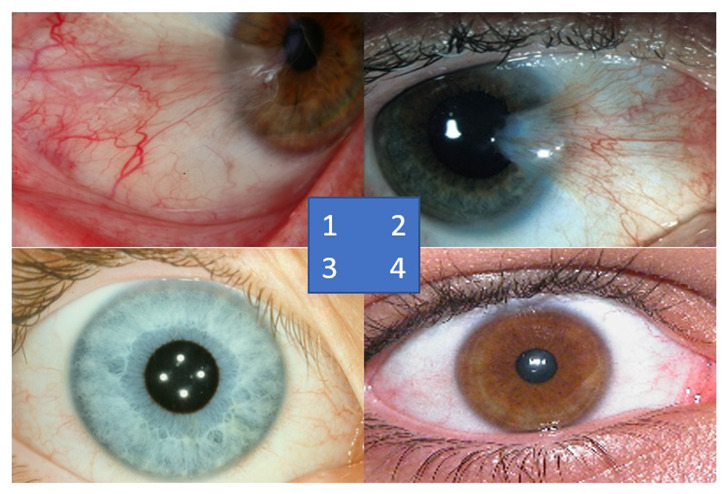
Sample of images from Zaki et al. [19] dataset; sample no. 1 is from the Australian Pterygium dataset, sample no. 2 is from the Brazil Pterygium dataset, sample no. 3 is from the MILES dataset, and sample no. 4 is from the UBIRIS dataset.

In Zaki et al. [19], the dataset was extracted from four different sources, which were UBIRIS, MILES, Australia Pterygium, and Brazil Pterygium. The two former sources represent normal eye cases, while the latter two sources represent pterygium cases. The dataset does not provide severity labels, but it consists of all pterygium stages from the trace to the severe cases. A total of 30 normal samples each were randomly chosen from the UBIRIS and MILES datasets, and 30 random samplings each were also extracted from the Australian Pterygium and Brazil Pterygium datasets. Hence, the total number of samples was 120 anterior segment photograph images with various resolutions. Table 2 shows the full information of the Zaki et al. dataset. This dataset intends to cover both blue and brown iris-colored eyes with both low- and high-resolution images. However, the samples for normal cases were captured in a more standardized condition with stable lighting and a straight gaze towards the camera. On the other hand, the lighting condition for the pterygium cases varies significantly from one sample to another, as some of the images were captured while the pupil was not directed straight at the camera. These variations between normal and pterygium cases might lead the feature extraction module to learn the setup condition rather than the overgrowth of tissues in detecting pterygium. The ground truth for this dataset was annotated independently by two optometrists, whereby all images were saved in Joint Photographic Experts Group (JPEG) format. Some samples of the dataset are shown in Figure 5.

Another classification dataset was collected by Fang et al. [22], with a total of 2106 images, which are further divided into training, validation, and testing subsets according to the ratio of 7:1:2. The authors have ensured that the sample distribution between normal and pterygium cases is almost equal; they were randomly pooled from a large source of 15,192 images. The data were originally collected by Singapore Epidemiology of Eye Diseases using two cameras, which were the Topcon model DC-1 and MEC-5-ASL-D7100-N85, and were also saved in JPEG format. Some of the samples were taken with a slit-lamp configuration, while some of them were captured from a hand-held configuration. The samples were taken from among three major ethnicities in Singapore, Chinese, Malay, and Indian, whereby the iris color is predominantly brown. The authors have added another class of referable pterygium, which is defined as mild and severe cases, in which the encroachment has intruded more than 2.5 mm into the corneal region. However, the number of samples is considerably low compared to the early-stage cases, with only 8.4% of the total samples. They have resized all images to 224 × 224 pixels to match the input requirement of the deep learning model that they have tested.

**Figure 6:**
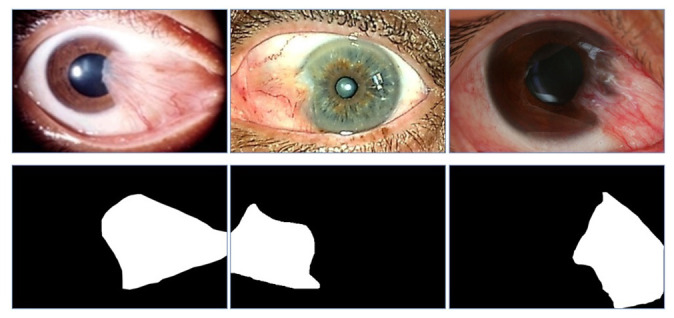
Samples of pterygium images and their corresponding ground truth semantic images. The first row of images are the original anterior segment photograph images and the second row of images are the corresponding ground truth label images.

Abdani et al. [7] focused only on the segmentation dataset. The images were sourced from the full Australian Pterygium classification dataset, which was collected by Professor Lawrence Hirst from the Australian Pterygium Centre. The total number of images in this dataset is 328 anterior segment photograph images with their corresponding semantic segmentation ground truth. The label was manually traced by the medical and biomedical researchers, and it is saved in JPEG format. They have followed the annotation protocol from the 2017 Automated Cardiac Diagnosis Challenge: Segmentation [24], whereby the bio-medical researcher will trace the pterygium tissues’ outline first, before the final boundary is finalized together with the medical researcher. Only two semantic classes are considered, namely whether the pixel belongs to the pterygium class or not. The original resolution of 4064 × 2704 is reduced to 450 × 300, whereby the user can down-scale the resolution further, independently, according to the deep learning model requirement. GIMP2 version 2.10.14 with the pencil tool was used to trace the pterygium tissue boundary. This dataset also contains cases from various severity levels of pterygium, which include trace, mild, moderate, and severe cases. Some of the pterygium samples and their corresponding ground truth labels are shown in Figure 6.

EyeHealer is a database dedicated to various eye diseases based on anterior segment photograph imaging [23]. It contains 3813 images of 23 diseases, with unbalanced sample distribution between the classes. The authors also provide the label for the semantic segmentation of the lesions for all diseases. Each of the images contains only a unique eye disease and, hence, one image corresponds to one disease only. The pterygium class has the second-highest number of samples, with 482 images, which were taken using a slit-lamp configuration with various types of camera. This dataset does not contain a healthy normal class and all diseases were initially validated by six ophthalmologists from the Zhongshan Ophthalmic Centre of Sun Yat-sen University and West China Hospital, before two senior ophthalmologists verified the labeling output. Any discrepancy is finalized through discussion between the two seniors. However, only 10 classes of diseases have a total sample of more than 100 images and, thus, training a deep learning model from scratch will be a difficult task. It is even more challenging to fit a deep learning semantic segmentation model for diseases with a low number of samples due to over-fitting issues. Moreover, no severity level information of the diseases is provided. In our opinion, this dataset is not a large-scale resource for training an automated eye system as the authors have claimed, although it provides a dataset for various eye diseases’ detection but with a low number of samples for most classes. The resolution of the image provided is also relatively small compared to the other datasets, with just 256 × 256 pixels, and images are also stored in JPEG format. For all datasets that were collected through slit-lamp anterior segment photograph mode, each image was captured when the respective subject had placed his whole head on a dedicated mechanism. Hence, the physical distance between the subjects and the camera remains relatively constant. As for the field of view, it varies according to the different types of camera used to capture the images. However, the lens has been set up optimally so that it will zoom directly into the eye region only, either the left or right eye. EyeHealer has used BX-900 slit lamps with a stereo angle of $13^{{}^{\circ}}$ with a maximum ocular magnification of 12.5. Pupil distance between the eyes is set at a minimum of 52 mm, which results in a field of view of 14.6 mm × 21.9 mm when the magnification level is set to unity. The spectral range of the captured image is 400 nm to 750 nm, which can be maximally rotated by $\pm 90^{{}^{\circ}}$. The focal length is 170 mm, whereby the captured slit image width and length have a continuous range of [0 mm, 8 mm] and [1 mm, 8mm], respectively. The camera can be operated by a single operator with a 3-dimension adjuster of length, height, and side. Besides this, for the deep learning-based test, the physical distance variation will not pose a difficult challenge to the classification and segmentation networks as some of the models are embedded with multi-scale capability and, hence, a wider range of physical measurement can be tolerated.

### 5.2. Conventional Approach to Automated Pterygium System

In the conventional approach of computer-assisted pterygium systems, basic computer vision algorithms and conventional machine learning techniques are used to deduce the presence of pterygium inside an image. The researchers need to handcraft the feature extraction method or select the best set of features that were used to represent the pterygium cases in order to distinguish them from normal healthy eyes. In [25], the authors have performed a reliability test on the basic pterygium grading system based on the redness value of the fibrovascular tissues. They have classified the redness levels according to three categories, which are atrophic, intermediate, and fleshy, based on Tan et al.’s [26] assessment protocol. They have collected eye images from 93 samples from both male and female participants with confirmed pterygium conditions. However, they also ensured that the patients, who are aged between 20 to 70 years old, did not have other eye conditions that might have affected the reliability of the test, such as ocular trauma or ocular surgery. The results, which were measured using intraclass correlation coefficients, showed that the reliability of the proposed method is on par with medical expert grading, with a small coefficient difference between them. Mesquita and Figueiredo [27] argued that the pterygium surgical procedure should only be performed for severe cases, as recurrence cases are normal if the benign tissues are removed at the early stage. Hence, they have designed a computer-assisted system to measure the encroachment of the benign tissues into the corneal region. A small-sized dataset of only 58 images was analyzed, whereby they extracted the iris region first by using circular Hugh transform, before the encroachment region was extracted from the segmented circular region. Therefore, their method is only applicable for the later stage of pterygium, since the early stages of pterygium do not display visible tissue encroachment onto the corneal region. A combination of Otsu thresholding and dilation operator was performed to extract the possible regions of interest, which resulted in a few fragmented detections. These regions were linked together using the connected components procedure to produce a single large lesion segmented map. Canny edge detector and Gaussian filter were also applied during the iris segmentation phase to produce a better circular map of the iris region.

In [28], Gao et al. have developed a pterygium detection system that is able to distinguish the condition compared to cataract cases. They have also focused on the later stage of pterygium, whereby only benign tissues inside the iris region were analyzed. Although the tested dataset comes in RGB format, they have utilized only the red channel and the transformed Fisher channel to extract the abnormal tissues based on the redness level assumption. Once the pupil region has been detected, a simple thresholding method is applied to extract the possible pterygium region. They have tested their proposed method on a large-sized dataset, but with a relatively low pterygium class of only 67 cases. Minami et al. [29] then analyzed the spherical property of the corneal region using Fourier series harmonic analysis. They identified six distinct uniform diameter values that ranged from 1 mm to 6 mm to represent pterygium advancement. Although their analyzer is able to measure a diameter up to 8 mm, they found that the higher values tend to be affected by eyelid disturbance, especially for older people, who tend to have droopy eyelids. In fact, their dataset consists of many senior patients, with an average age of 67 years old, taken from among 456 primary pterygium patients. They have performed statistical linear regression analysis to verify their findings, whereby they have proven the correlation between corneal irregularity and pterygium tissue advancement.

A simple artificial neural network algorithm (ANN) was implemented in [30] to compare the grading performance of pterygium conditions between medical practitioners and a machine learning system. Two medical practitioners were involved in this study, whereby one practitioner was a junior ophthalmologist with less than 3 years of experience and the other one was a senior ophthalmologist with more than 5 years of experience. The main goal of their study was to classify an image into three classes of pterygium severity level, which were annotated based on the redness level of the overgrowth tissues. The ANN was trained for 300 epochs with a low number of data. A total of 68 slit-lamp images were divided equally into training and test subsets, which resulted in low training data. The network architecture was also relatively small, with a single hidden layer of 10 nodes. Five handcrafted features were extracted from four different color spaces of RGB, YUV, HSI, and CIE XYZ. Although the authors claimed that the machine learning graders performed on par with the medical practitioners, the results are questionable as the validation data were too low to come to a viable conclusion. A more comprehensive set of features was studied in [19], which was in line with the previous study’s approach, whereby the encroachment of the pterygium tissues into the corneal region was the primary focus. They proposed a four-step approach through image enhancement, corneal region segmentation, feature selection, and classification modules. In the image enhancement module, the RGB color space is converted to HSV space through the HSV-Sigmoid image enhancement method, which is followed by edge operator manipulation to extract the corneal region. Four sets of handcrafted features were used as the input to the classification module, which included circularity, Haralick’s circularity, eccentricity, and solidity. Two types of classifier were tested, which were support vector machine (SVM) and ANN. Three variants of ANN that differed according to the number of hidden nodes were tested, while four variants of SVM were also tested that differed according to the kernel type. A total of 120 images from four different sources were used to validate the performance, with 60 images each for the pterygium and normal classes. The best performance was achieved by the SVM classifier, with a unity standard deviation of radial basis function (RBF) kernel. However, the ANN classifier produced better average specificity performance compared to the SVM-RBF classifier.

Jais et al. [31] focused more effort on the design of the classification module by testing four different types of classifier. There were five input attributes or features used to represent the pterygium condition, which were redness, thickness, length, total area, and dry weight, which were taken from 93 samples. There were no pre-processing or image enhancement procedures applied to the original input data. The four classifiers that were tested were decision tree, SVM, logistic regression, and Naive Bayes, whereby the best performance was obtained by the SVM classifier. They also analyzed the ensemble architecture of the proposed classifiers with boosting and bagging procedures to better train the networks. However, still the problem of low training data persists and might limit the ability of the classifier in learning the optimal hyperplane. This assumption is also supported by their results as the bagging, boosting, and ensemble procedures did not increase the classification performance of the pterygium cases. Rather than focusing on the classification task, the work in [32] used simple image processing algorithms to extract the size of the pterygium tissues in the corneal region. The authors manually annotated the circular region that surrounds the limbus, and they then smoothly marked the pterygium tissue areas. This assumption results in a less accurate region of interest division as the encroached tissues are not usually divided smoothly and, hence, it has become the motivation of several researchers to perform semantic segmentation to extract the exact lesions of interest [7,33]. Table 3 shows a summary of the conventional machine learning approach to pterygium detection and localization.

The primary weakness of the conventional methods lies in the need for handcrafted features, whereby the optimal set will be manually formulated given the different circumstances. In [27,28], the images were captured in a low-resolution format, whereby not many patterns could be discerned from a human observer’s perspective. Although the work in [19] used a mixture of low and high resolution, still the quality division between pterygium and non-pterygium is too obvious. The healthy eyes were captured at a relatively higher resolution compared to the pterygium cases. Similarly, the works in [31,32] only used subjects from Asian countries, which limits the variety in iris color. Hence, these systems will not be robust enough to cater to mixed-race situations. Finally, all these conventional experiments were validated by a relatively low number of samples per class, except for the Minami et al. [29] and Gao et al. [28] works. Furthermore, many of these experiments were trained and tested with less than 150 samples, which is far from the current deep learning standard, which uses more than 1000 samples per class.

### 5.3. Deep Learning Approach to Automated Pterygium System

In the deep learning approach, the features are optimally extracted through the training process, rather than being selected by the model’s designer. The feature extraction module is usually coupled with the classification/segmentation module, which is then optimally trained in an end-to-end procedure to obtain the best set of features to represent the problem of interest. Hence, in the case of pterygium screening and diagnosis, the input image will be passed to the networks, whereby three problems of interest will be analyzed, which are pterygium classification, pterygium tissue localization, and pterygium tissue semantic segmentation. During the early adoption of deep learning techniques for pterygium screening, the network was designed to be compact in nature [34,35]. In [34], Lopez and Aquilera designed compact deep learning networks with only a single layer of convolutional neural networks (CNN) and two dense feed-forward layers. The network was down-pooled and flattened before being passed to the dense classifier. They used Zaki et al.’s dataset [19] with additional explicit data augmentation through Gaussian blurring and rotation transformation to validate their proposed networks. The augmented data were added to balance the training data distribution between normal and pterygium cases. They analyzed two types of input data, either three-channel RGB or a single channel of grey-scale images, both with an input resolution of 150 × 150 pixels. No pre-trained weights were utilized and the best classification performance was obtained by the RGB version of the networks. A single dropout unit [36] was added immediately after the convolutional layers to reduce the likelihood of over-fitting. Another compact architecture was proposed in [35] with two layers of convolutional networks and two layers of dense feed-forward connections. The design of the CNN layers followed the first two layers of the VGG-M architecture [37] so that the pre-trained weights could be used to initialize the model. They tested its performance on the same dataset as the previous paper, but no data augmentation was performed besides only the transfer learning of the weights. They analyzed several configurations of network regularization, which included local response normalization, batch normalization, and dropout. Their best accuracy of 98.33% was obtained by using embedded local response normalization and dropout layers, which is a significant improvement compared to Lopez and Aquilera’s [34] accuracy of 93.5%.

Instead of focusing on the compact version of the network, Zheng et al. [38] focused on the lightweight analysis of the network, whereby the model size is the main concern. They focused on a three-class problem of normal, observed pterygium, and surgery-required pterygium. The last class involved cases in which the benign tissues had encroached onto the pupil region. They collected their own dataset, with a total of 436 images, with all patients having brown-colored irises. Both versions of the lightweight MobileNet [39] were tested, which was benchmarked with other popular deep models, including AlexNet [40], VGG-16 [41], and ResNet-18 [42]. The MobileNet reduces the memory usage by implementing a factorized version of convolution, which is reduced to a combination of depth-wise and point-wise convolution operations. The authors found that MobileNet with data augmentation produced the best overall results, and the lowest classification performance was returned for the second class, which was the observed pterygium class. Their dataset was relatively small for training the MobileNet architecture optimally and they also opted to use the small version of ResNet. This choice is understandable because of the limited training data, whereby a deeper model will usually experience an under-fitting problem. In fact, the usage of single-race patients resulted in a homogeneous iris color and reduced the robustness capability of the network. Another work that analyzed the performance of the VGG-16 architecture for pterygium classification was proposed by Fang et al. [22]. The uniqueness of their method is that the validation process was tested on both slit-lamp and hand-held eye imaging. All the previous methods used the slit-lamp mode of capturing the anterior segment images, which produces fewer variations compared to hand-held imaging. Although the total number of data was high, the tested pterygium cases were relatively few, with only 217 images. Moreover, they further divided the pterygium cases into observed pterygium and surgery-required subsets. The accuracy performance for slit-lamp and hand-held imaging was high, with 99.1% and 99.7%, respectively. However, it was observed that the specificity performance for the hand-held cases was lower compared to the slit-lamp mode. The lower performance can be attributed to more angle variations in capturing the eye images, as well as variations in background lighting.

A deeper network can be observed in the work by Xu et al. [43], whereby EfficientNet is used to classify anterior eye images to identify the cases of observed and surgery-required pterygium. EfficientNet-B6 [44], which is the second-deepest network from the EfficientNet family of architectures, was trained by using a total of 750 images, and an additional 470 images were used during the validation phase. The images were obtained from [22], which was collected from the Affiliated Eye Hospital of Nanjing Medical University. The same result pattern could be observed between their work and the work by Fang et al., whereby the accuracy is the highest for the normal cases and the accuracy is the lowest for the observed pterygium cases. For the observed pterygium cases, the benign tissues are not clearly visible and, hence, some confusion might arise between this class and the other two classes. Pterygium-Net [4] is a network of three convolutional layers with three dense feed-forward layers that are specifically designed for pterygium classification and localization. The authors designed the pterygium tissue localization system by using an object detection-based methodology through bounding box representation. Candidate boxes of various sizes and locations were sampled randomly according to the Gaussian distribution throughout the eye image, and each box will be assigned a likelihood of containing pterygium tissue. The Hanning window was also applied to give more weight to the middle part of the candidate boxes, whereby the weights will gradually decrease as it moves further away from the center of the box. They also explored network variations from one to five convolutional layers, whereby three CNN layers produced the best detection for both classification ad localization tasks. The localization output was finalized according to the average value of the top-*n* candidate boxes; as such, the middle points, width, and height of the output box are the results of mean values derived from the top-*n* candidate boxes. Their method’s weakness can be observed if the pterygium tissues are slender in shape, whereby the box representation will incorrectly capture the region of the lesion compared to the semantic segmentation approach.

Taking motivation from the previous method’s weakness, the work in [33] approached the problem of pterygium tissue localization through a semantic segmentation methodology. Hence, a more accurate representation of the tissues can be better obtained as the output will be pixel-based labeling. In [33], the authors have proposed dense feed-forward connection addition to the original DeepLab V1 [45] and Deeplab V2 [46] architectures by concatenating a skip connection within each convolutional block. They have annotated a total of 328 images from the original Australian Pterygium dataset. The dense connection has managed to increase the intersection over union (IoU) performance for both models. Dense DeepLab V1 produced an IoU of 0.8250, compared to the original DeepLab V1, with an IoU of 0.8004. Meanwhile, the dense feed-forward addition to the DeepLab V2 only managed to increase the IoU slightly from 0.8381 to 0.8327. Hence, the contribution of the dense feed-forward connection for DeepLab V1 is more significant compared to DeepLab V2. Another improvement in the semantic segmentation of pterygium tissues was proposed in [7] by introducing group and shuffle units to the segmentation networks. The performance improvement was significant, whereby the best variant returned an IoU of 0.8640. The base network was derived from FC-DenseNet [47] with symmetrical encoder and decoder modules. The group and shuffle unit was added to replace the first convolutional layer of each block for both the encoder and decoder sides. This unit addition reduces the likelihood of having a single set of dominant features, whereby the networks are forced to learn from shuffled input streams. Moreover, the authors added a spatial pyramid pooling [48] unit at the bottleneck layer to increase the multi-scale capability of the network. This work still relies on gradual down-pooling steps, whereby the feature map size is reduced as more convolutional layers are added and some information will be lost during the down-sampling, which is in contrast to the recent methodology of the high-resolution semantic segmentation approach [49]. Another paper by Cai et al. [23] also explored several existing deep learning semantic segmentation models for locating pterygium lesions. They analyzed four models, which were DRUnet [50], SegNet [51], PSPNet [48], and DeepLab V3 [52]. The first two models represent the symmetrical encoder–decoder network architecture, while the last two models represent the asymmetrical encoder–decoder network architecture. Their results, which were collected on 482 slit-lamp images, indicate that DeepLab V3 produced the best performance for pterygium lesion detection.

In summary, the work by Lopez and Aquilera [34] has a clear weakness in terms of the quality of the extracted features due to the utilization of a single CNN layer only. On the flip side, it has a very low computational requirement due to the compact architecture with a low number of parameters. Meanwhile, the work in [4] not only introduced the bounding box localization approach to the infected areas, but it also used a transfer learning approach for initializing the CNN weights and biases. However, the utilization of the local response normalization layer reduced the model’s computational speed due to the low parallelization capability of the architecture. The slow computational speed issue is overcome by Zheng et al. [38] through a lightweight model that has implemented a factorized convolution scheme, which happens to reduce also the memory usage. However, the proposed MobileNet architecture was only trained by using a low number of samples without any pre-trained weights. A similar issue was also encountered in the works in [22,43], whereby the authors used a low number of samples and, even worse, the dataset distribution was imbalanced between the classes. Furthermore, the tested data were relatively homogeneous, being derived from a single ethnicity only. In [33], the first semantic segmentation approach to automatically extract the boundaries of the infected areas has been introduced by promoting the DeepLab models. They have embedded a set of dense connections to carry over more information between the deep layers. However, the main weakness of this approach is that it requires a large amount of memory as the number of channels will grow significantly due to the usage of the concatenate operator, which combines the incoming layers and the existing output layers. A fine-tuned model was introduced in [7] by introducing the group and shuffle layers, whereby the model was found to be more capable of learning from diverse input streams for better segmentation accuracy. However, the model capability will be severely affected if the number of groups is not properly optimized since the number of filters per group might become too small. On the other hand, the main advantage of Cai et al.’s [23] work can be traced to the usage of a large number of data for several eye disease classes, which will enable a more comprehensive screening system. However, its main weakness can be observed through the standard utilization of existing deep models, without any model optimization. In addition to this, no transfer learning scheme has been applied, which may pose an issue in training a deep segmentation network of various eye diseases. Table 4 shows a summary of the deep learning approach to pterygium detection and localization.

## 6. Conclusions and Future Works

This paper has summarized the overall state-of-the-art techniques in automated pterygium screening systems, which usually consist of two main tasks: either classification or segmentation. Pterygium at the early stage is not a harmful condition, but at the later stage, it will encroach towards the corneal region and eventually the pupil region, which will affect the patient’s eyesight. Hence, it is important to diagnose the pterygium condition at the early stage, so that mitigative procedures can be administered to prevent it from becoming worse. However, most of the pterygium patients are not aware of the condition, which may be related to one of its risk factors, which is a low educational level. Therefore, an automated screening system using a hand-held camera will enable patients to perform self-screening frequently, without any substantial cost. In addition to this, the accuracy of pterygium screening has improved significantly with the implementation of deep learning methods, compared to the conventional machine learning methods. This improvement can be observed even for the lesion segmentation task, whereby the lesions of interest can be directly extracted to determine the severity level. Furthermore, the number of available datasets for training the deep learning methods has also increased during the past few years. In conclusion, the research in automated pterygium screening systems has significantly advanced, with good screening performance that has been validated on various datasets.

Even with the current state-of-the-art systems, the performance of an automated pterygium screening system can be further developed by addressing the following research directions:

1 Development of a comprehensive lesion dataset that can be used to determine the severity level. The current research focuses on identifying the severity level, without performing exact measurement of the lesions’ encroachment onto the corneal region. Even with the combination of the dataset from both Abdani et al. [33] and Cai et al. [23], the total annotated lesion data amounts to only 810 images, which is far from the ideal number of training data.
2 Data augmentation through synthetically generated images using generative adversarial network (GAN). At present, none of the research has implemented GAN to augment the training data. Based on past research, only the explicit transformation of the original data through rotation and blurring functions has been implemented to increase the number of training data. The synthetic data can be generated according to the specific label by using conditional GAN to balance out the number of training data between various classes [53].
3 Integrate an attention mechanism into the classification and segmentation networks, whereby the lesions are normally observed at certain locations. The likelihood of pterygium tissue to originate from the medial canthus is also higher compared to the lateral canthus, which indicates that certain regions should be emphasized more compared to others. The attention mechanism will allocate more weight towards specific locations on the image and, hence, increase the likelihood of accurate classification and segmentation.

## Figures and Tables

**Table 1:** Summary of the risk factors of the pterygium condition.

Study	Publication Year	Sample Size	Study Location	Risk Factors
West and Munoz [14]	2009	4774	Arizona, USA	Low income, low educational status, and exposure to sunlight
Cajucom-Uy et al. [12]	2010	3282	Singapore	Increasing age, male, outdoor occupation, and systemic factors
Zhong et al. [8]	2012	2133	Dali, China	Increasing age, lack of formal education, and outdoor occupation
Jiao et al. [9]	2014	17,816	Shangdong Province, China	Older age, outdoor time, educational level, and usage of sunglasses
Malefikar et al. [11]	2017	420	Ilam Province, Iran	Family history of pterygium, cigarette smoking, history of baking, age, and severe blepharitis
Wang et al. [10]	2020	2651	Inner Mongolia, China	Age, outdoor occupation, and time spent in rural areas
Fekadu et al. [13]	2020	400	Gambella, Ethiopia	Male, outdoor occupation, and exposure to sunlight

**Table 2:** Summary of the Zaki et al. [19] pterygium dataset.

Sources	No. of Samples	Resolution	Format	Iris Colors
Australian Pterygium	30	4064 × 2704	JPEG	Blue and Brown
Brazil Pterygium	30	308 × 231	JPEG	Blue and Brown
MILES	30	1747 × 1180	JPEG	Blue
UBIRIS	30	200 × 150	JPEG	Brown

**Table 3:** Summary of automated pterygium screening systems using conventional approach.

Study	Task	Sample Size	Strength	Weakness
Hilmi et al. [25]	Severity grading	93 pterygium images	Three-class problem; atrophic, intermediate, and fleshy	Relies only on redness information
Mesquita and Figueiredo [27]	Tissue growth progress	58 pterygium images	Good segmentation even if the iris and pterygium tissues look similar in color	Circular Hugh transform only works if the gaze is perpendicular to the camera
Gao et al. [28]	Classification: pterygium and non-pterygium	30 pterygium images and 854 non-pterygium images	Utilizes unique Fisher channel	Too many deterministic thresholds, which will not work when tested on different iris colors
Minami et al. [29]	Tissue growth progress	456 pterygium images	Fourier frequency analysis to represent the growth ring of pterygium tissues	Only six quantized levels to represent the tissue growth
Azemin et al. [30]	Severity grading	68 pterygium images	Utilizes compact ANN with five features as input	Relies heavily only color information without looking at pterygium tissue textures
Zaki et al. [19]	Classification: Pterygium and normal	60 pterygium images and 60 healthy eye images	Gradient-based lesion extraction, which is robust to various iris colors	Their dataset is skewed, whereby the healthy data were captured in a more standardized condition
Jais et al. [31]	Severity grading	93 pterygium images	Analyzes multiple conventional machine learning classifiers	No cross-validation, test dataset comprises only 9 images
Radzi et al. [32]	Lesion segmentation	120 pterygium images	Introduces pixel-based ratio between lesions and non-lesions to determine severity level	Smooth lesion boundary, which is not accurate for most cases

**Table 4:** Summary of automated pterygium screening systems using deep learning approach.

Study	Task	Sample Size	Strength	Weakness
Lopez and Aquilera [34]	Classifi-cation	325 pterygium images and 2692 healthy eye images	Perform data augmentation to balance training dataset	A single convolutional layer only
Abdani et al. [35]	Classifi-cation	60 pterygium images and 60 healthy eye images	Analyze various regularization methods and implement transfer learning	Trained using low total number of data
Zheng et al. [38]	Classifi-cation	142 normal images, 144 observed pterygium images, and 150 surgery-required images	Lightweight deep model using MobileNet architectures	Training data are relatively low for training the MobileNet effectively
Fang et al. [22]	Classifi-cation	Test data: 217 pterygium images and 6094 healthy eye images	Tested on both slit-lamp and hand-held images	Dataset severely imbalanced with small number of pterygium cases
Xu et al. [43]	Classifi-cation	189 pterygium images, 171 observed pterygium, and 110 surgery-required images	Implement state-of-the-art EfficientNet architecture	Lowest detection for observed pterygium class, even though tested on brown iris color dataset only
Pterygium-Net [4]	Locali-zation	60 pterygium images	Locate the region of pterygium lesions	Bounding box representation is not suitable for slender-shaped tissues
Abdani et al. [33]	Segmen-tation	328 pterygium images	Embed dense feed-forward layer to DeepLab architecture	Dense connection for DeepLab V2 only improves the performance slightly
Abdani et al. [7]	Segmen-tation	328 pterygium images	Embed group and shuffle unit with multi-scale parallel networks	Available dataset is relatively small for complex deep learning architecture
EyeHealer [23]	Classifi-cation and Segmentation	482 pterygium images	Compare with various eye disease	Low number of training data except for cataract and pterygium cases

## Data Availability

Not applicable.

## Abbreviations

The following abbreviations are used in this manuscript:

IoU	Intersection over Union
CNN	Convolutional Neural Networks
SVM	Support Vector Machines
ANN	Artificial Neural Networks
GAN	Generative Adversarial Networks
JPEG	Joint Photographic Experts Group
